# A Coarse Grained Model for a Lipid Membrane with Physiological Composition and Leaflet Asymmetry

**DOI:** 10.1371/journal.pone.0144814

**Published:** 2015-12-14

**Authors:** Satyan Sharma, Brian N. Kim, Phillip J. Stansfeld, Mark S. P. Sansom, Manfred Lindau

**Affiliations:** 1 Laboratory for Nanoscale Cell Biology, Max-Planck-Institute for Biophysical Chemistry, Göttingen, Germany; 2 School of Applied and Engineering Physics, Cornell University, Ithaca, New York, United States of America; 3 Department of Biochemistry, University of Oxford, South Parks Road, Oxford, England, United Kingdom; University of Leeds, UNITED KINGDOM

## Abstract

The resemblance of lipid membrane models to physiological membranes determines how well molecular dynamics (MD) simulations imitate the dynamic behavior of cell membranes and membrane proteins. Physiological lipid membranes are composed of multiple types of phospholipids, and the leaflet compositions are generally asymmetric. Here we describe an approach for self-assembly of a Coarse-Grained (CG) membrane model with physiological composition and leaflet asymmetry using the MARTINI force field. An initial set-up of two boxes with different types of lipids according to the leaflet asymmetry of mammalian cell membranes stacked with 0.5 nm overlap, reliably resulted in the self-assembly of bilayer membranes with leaflet asymmetry resembling that of physiological mammalian cell membranes. Self-assembly in the presence of a fragment of the plasma membrane protein syntaxin 1A led to spontaneous specific positioning of phosphatidylionositol(4,5)bisphosphate at a positively charged stretch of syntaxin consistent with experimental data. An analogous approach choosing an initial set-up with two concentric shells filled with different lipid types results in successful assembly of a spherical vesicle with asymmetric leaflet composition. Self-assembly of the vesicle in the presence of the synaptic vesicle protein synaptobrevin 2 revealed the correct position of the synaptobrevin transmembrane domain. This is the first CG MD method to form a membrane with physiological lipid composition as well as leaflet asymmetry by self-assembly and will enable unbiased studies of the incorporation and dynamics of membrane proteins in more realistic CG membrane models.

## Introduction

Coarse-Grained (CG) molecular dynamics (MD) simulations have become an important tool to study the insertion and dynamic behavior of membrane proteins in lipid membranes. Generally, the membrane models used in MD simulations to study membrane properties [1, 2], lipid rafts [3], membrane fusion [4], protein insertions and lipid-protein interactions [5] consist of only one- or two-types of lipids. The lipid composition should be considered carefully because it determines the physical properties, such as thickness, area per lipid (APL), bending modulus, and curvature of the membrane [1, 2]. In addition, lipid-protein interactions that are important determinants of the dynamic behavior of membrane proteins within the membrane critically depend on the lipid types and their localization in the membrane. Thus, it is important to choose an accurate membrane model in membrane-proteins simulations to derive relevant conclusions.

### Leaflet asymmetry of synaptic vesicle membrane

Synaptic vesicles (SV), that play a key role in synaptic transmission, incorporate numerous different proteins in their membrane, including fusion-mediating SNARE proteins, transporters and channels [6]. A realistic CG model of SV membranes would provide opportunities to study SV membrane proteins in MD simulations that provide sub-nm and femto-sec resolution over extended periods of time, towards a better understanding of the function of trafficking proteins, transporters and channels. Such a model should, first of all, be composed of the types of lipid as determined experimentally in SV membranes [6], which are from most to least abundant: phosphatidylethanolamine (PE), phosphatidycholine (PC), phosphatidylserine (PS), sphingomyelin (SM), and other lipids (< 4%) including phosphatidylinositol and hexosylceramide. In addition to phospholipids, cholesterol is highly abundant in SV membranes and affects membrane permeability, stiffness, and thickness [1, 2].

Even though lipids in membranes are in a fluid-like state and exhibit rapid lateral diffusion, the lipid flipping between the opposite leaflets is mediated in a controlled fashion [7, 8], giving cells control over the lipid compositions of each leaflet separately. The asymmetric composition of plasma membrane was determined experimentally [9, 10], and its importance in many cellular functions was studied [11–13]. Since the SV is a recycling organelle, its leaflet asymmetry is expected to reflect that of plasma membranes with the cytoplasmic (CP) leaflet mainly composed of PE and PS and the extracellular (or intravesicular) leaflet mainly composed of PC and SM [10, 14]. Interestingly, cholesterol was also reported to maintain a somewhat asymmetric distribution, preferably located in the CP leaflet [15–17] of plasma membranes, despite of its rapid flipping rate [18]. We chose a mixture of Palmitoyl-Oleoyl-PC (POPC), -PE (POPE), -PS (POPS), palmitoyl-SM (PSM) and cholesterol [3, 19, 20] in ratios to approximate the major components of SV lipid membranes in a CG-MD model.

### Self-assembly of membrane and membrane-protein

Recently, to imitate the complexity and asymmetry of membranes, several CG membrane models have been developed. These include, for example, a model of a generalized plasma membrane [21], models of thylakoid membranes from cyanobacteria and higher plants [22], models of stratum corneum [23, 24] and red blood cell (RBC) plasma membrane [25]. The building of an asymmetric membrane model is a challenge due to the difficulty in assigning the number of lipids in each leaflet of the asymmetric bilayer. In these previous studies the initial configurations of membranes were built either by simply replacing a fraction of the lipids in a single component membrane by the lipids of choice, either keeping the number of lipids in each leaflet of the membrane constant or choosing the number of different lipid types in each leaflet based on a prior estimation of APL for the different lipid types. Nearly all of the procedures currently available in literature, such as CHARMM-GUI [26], MemGen [27], INSANE (INSert membrANE) CG building tool [28]-to list a few; depend upon *a priori* assumption of the APL to choose the number of lipids in each leaflet. It is well known that the APL varies considerably in lipid mixtures, and thus symmetric bilayer simulations with various lipid mixtures are usually performed to determine the number of lipids required for the asymmetric bilayer leaflets. Therefore, there is a need for a method to generate asymmetric bilayers by self-assembly, which has the advantage of rapid, unbiased membrane formation independent of a priori APL assumptions.

**Fig 1:**
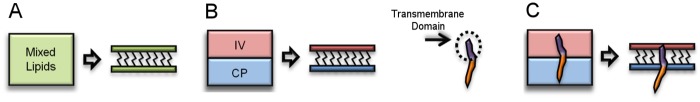
Methodology of membrane self-assembly. (A) The self-assembly of evenly mixed lipids result in a membrane with symmetric leaflet. (B) two stacked boxes filled with different lipids for each intravesicular (IV) and cytoplasmic (CP) leaflet self-assembles in an asymmetric membrane. (C) The same method can be used in simulations of membrane-protein insertion by including the membrane protein in the initial set-up the self-assembly. The transmembrane domain (in purple) of the protein aligns itself with the hydrophobic core of the membrane.

Spontaneous formation or self-assembly of membranes is one of the preferred methods to generate lipid bilayers in MD simulations [29], because it is largely unbiased and allows the system to find its free energy minimum. Such self-assembly simulations typically start from a simulation box of lipids randomly positioned and mixed with water. During the simulation, a spontaneous formation of a membrane occurs driven by hydrophobic effects of lipids in an aqueous environment [30], resulting in a symmetric membrane (Fig 1A). The self-assembly of membranes can be applied to membrane-protein simulations by inserting the protein in the initial set-up of the simulation box (before self-assembly), allowing the molecular dynamics simulation to reveal the position of the protein in the self-assembled proteolipid membrane. This method accurately predicts the protein position in the membrane [29, 31], but has so far only been applicable for membranes with symmetric leaflets. In contrast, protein insertion into a pre-assembled membrane requires an ad-hoc guess for its position that may not be near the energy minimum. The simulation may correct the position but this could take a long time if the protein is trapped in an undesired local energy minimum. Here we describe a simulation method to form a membrane with asymmetric lipid distributions in the two leaflets by self-assembly from an asymmetric initial set-up where the lipid mixtures desired for the two different leaflets are separated (Fig 1B). This method extends the study of protein insertion by self-assembly simulations to membranes with asymmetric leaflets.

## Results

To determine if any leaflet asymmetry is naturally produced in a mixture of different types of lipids as a result of their properties and their interactions with each other and the surrounding water molecules, initial simulations were performed using a conventional set-up with randomly mixed lipids (symmetric composition). As shown in Fig 1A, a bilayer formed during the simulation (200 ns) by self-assembly. However, as expected, the lipid composition in the two leaflets did not exhibit any significant asymmetry.

### Self-assembly of asymmetric membranes

In order to generate an asymmetric membrane, we focused on the initial arrangement of lipids in the simulation box, prior to the simulation so that the self-assembly may result in an asymmetric membrane. To this effect, two layers of randomly distributed lipids were placed on top of each other. The compositions of the top, cytoplasmic (CP), and the bottom, intravesicular (IV) leaflets were based on the published lipid composition of synaptic vesicles [6] and leaflet asymmetry of neuronal membranes [10]. The ratio of different lipids inserted into the simulation box resembled the physiological ratio to the best approximation possible (Table 1).

**Table 1:** Lipid composition of synaptic vesicle.

Lipids	% Physiological from [6]	% Membrane model	Number of molecules
PSM(PPCS)	7.4	7.4	36
PC(POPC)	36.1	36.8	180
PS(POPS)	12.3	12.9	63
PE(POPE)	41.9	42.9	210
CHOL	81.0	81.8	400

Numbers are in percentage of each phospholipid over the total number of phospholipids.

**Fig 2:**
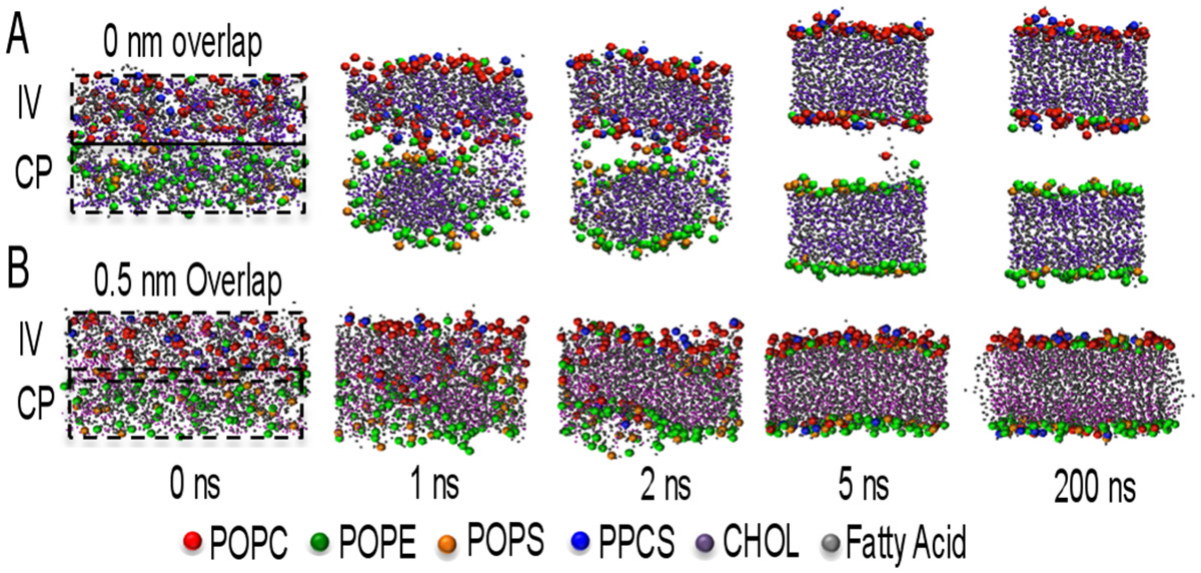
Overlapping of IV and CP lipids that self-assemble to an asymmetric membrane. IV lipids are mainly consisted of POPC and PPCS, and CP lipids are consisted of POPE and POPS. (A) The self- assembly with 0 nm overlap, in between IV and CP lipid boxes, separates into two membranes. The water molecules inserted (invisible) in between the gap of IV and CP prevents two boxes of lipids aggregating into a single membrane. But (B) in 0.5 nm overlapping, the center of IV + CP boxes is dense in lipids which prevents excessive water insertion leading to an asymmetric membrane with IV and CP leaflet. In both type of simulations the spontaneous formation occurred in 5 ns.

When these two lipid boxes were stacked directly on top of each other in a larger simulation box (Fig 1B, blue and red boxes) followed by addition of water and cations as required to compensate the negative net charge introduced by PS lipids, the self-assembly of a single bilayer frequently failed. Instead, the simulations could result in formation of two separate membranes (Fig 2A) or trapped water bubbles inside the membrane. These outcomes were due to the reduced density of lipids and increased density of water in the contact zone between the CP lipids and the IV lipids at the beginning of the simulation, which is caused by the way the lipid insertion of GROMACS worked. Since the procedure does not allow any molecules to extend beyond the edges of their box, it limits the orientation of lipids at the edges, thus reducing the density of lipids at the boundary of two boxes. During the water insertion, this boundary with fewer lipids was filled with increased density of water molecules compared to the center of either CP or IV boxes. To avoid the separation of CP and IV lipids during self-assembly, the CP and IV lipid boxes were stacked into the larger simulation box with a 0.5 nm overlap (Fig 2B). With this method, the lipid density in the contact zone was increased and self-assembly of asymmetric membrane was routinely successful.

Starting from two different random distributions of lipids, five self-assembly simulations were carried out for each initial configuration. All simulations resulted in successful formation of a planar bilayer, with asymmetric lipid distribution that was sustained during the remainder of the simulation time. During self-assembly, some lipids from the IV box ended up in the CP leaflet and some from the CP box in the IV leaflet. This limited mixing is advantageous because in a physiological membrane the lipid leaflet asymmetry is not 100%, rather there exists a strong preference for certain lipids to locate to a particular leaflet. For example, in neuronal membranes 90% of sphingomyelin is located in the extracellular leaflet and 10% on the cytoplasmic leaflet) [10]. When all PPCS molecules were initially placed in the IV box, 82% of these ended up in the IV leaflet after self-assembly. The asymmetry of the self-assembled membranes closely resembles the physiological values (Table 2).

**Table 2:** Lipid ratios of leaflet asymmetry in bilayer.

Lipids	Physiological from [12]	Asymmetric (0 ns)	Asymmetric (200ns)	Without Cholesterol (200 ns)	Symmetric (200 ns)
PSM(PPCS)	90	100	81.7 ± 3.7	75.9 ± 8.5	50.9 ± 5.8
PC(POPC)	89	100	78.7 ± 2.1	72.4 ± 3.6	49.1 ± 2.6
PS(POPS)	4	0	38.4 ± 4.4	32.3 ± 10.2	60.8 ± 5.1
PE(POPE)	15	0	32.2 ± 2.0	28.7 ± 4.8	43.7 ± 2.0
CHOL		36	46.2 ± 1.9	-	52.2 ± 1.1

Numbers are in percentage of each lipid in IV over its total number in the bilayer (IV+CP).

In addition to the major phospholipids PC, PS, PE, and sphingomyelin, synaptic vesicle membranes have a high cholesterol content. Cholesterol was therefore also included in the simulations in a cholesterol:lipid ratio taken from [6]. Inspired by previous reports on asymmetric cholesterol distribution in plasma membranes [15–17], cholesterol was distributed asymmetrically in the CP and IV boxes at the start of the simulation. However, the asymmetry of cholesterol was not preserved but equilibrated quickly during self-assembly to similar numbers in the two leaflets of the bilayer, with some cholesterol molecules found in the membrane center, as expected from previous reports on rapid flipping rates for cholesterol [18]. It therefore appears that asymmetric cholesterol distributions between the leaflets of a cell membrane must be generated and maintained by an active cellular mechanism.

### Physical properties of asymmetric membrane

**Fig 3:**
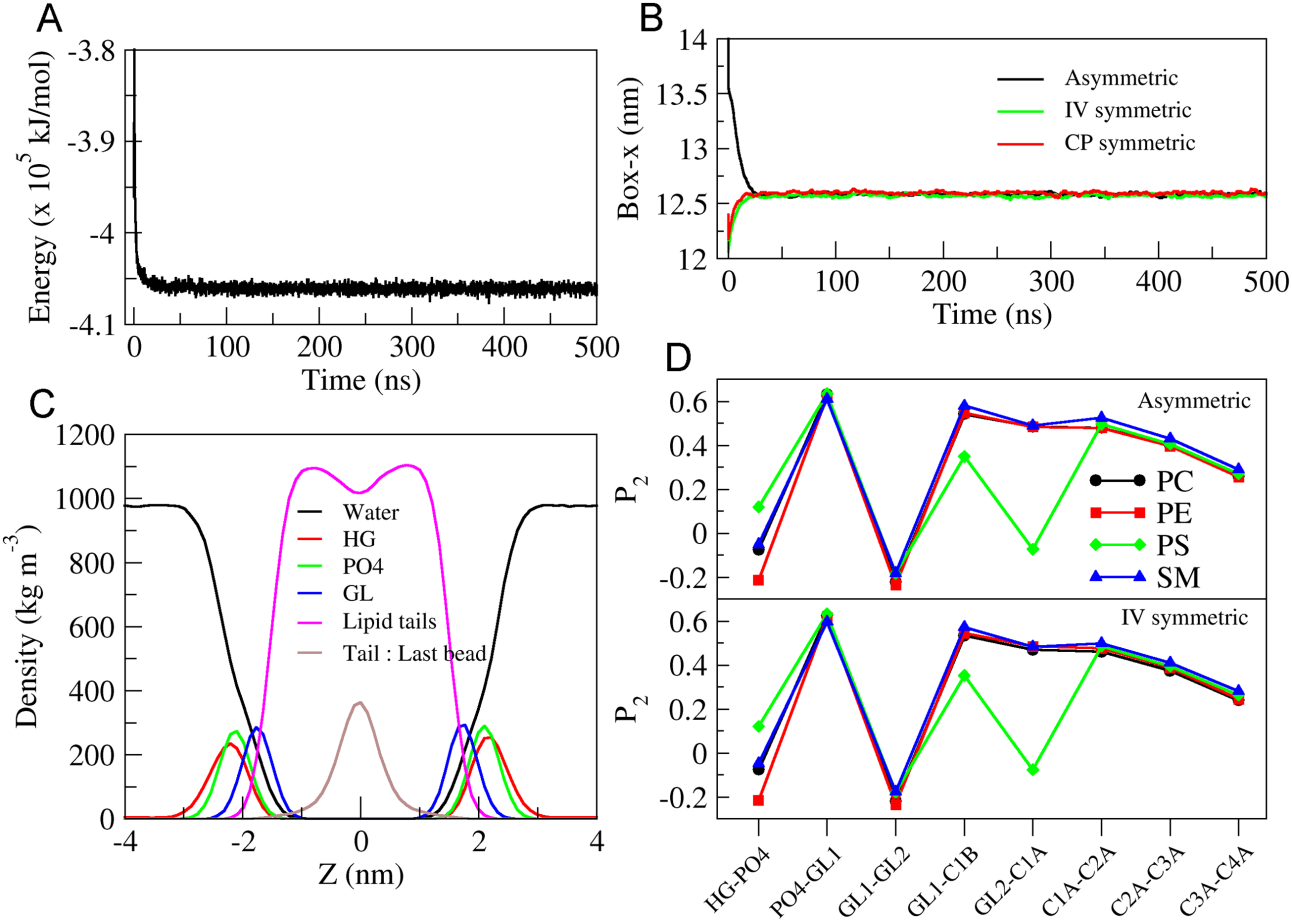
Structural characteristics of the cholesterol free membranes. (A) Potential energy profile over the simulation length for the asymmetric bilayer. (B) Box dimension, along x-axis, over time for asymmetric membrane (black) and symmetric membranes (see text) (IV-green, CP-red). (C) Partial density profiles of water (black) and various lipid moieties (HG—head group in red, PO4 in green, GL—glycerol backbone in blue, hydrophobic lipid tails in pink and terminal tail bead in brown) of the asymmetric bilayer along its normal (z-axis). The bilayer center is located at 0 nm. (D) Second-rank lipid order parameter for consecutive bonds of various lipids with respect to the bilayer normal. Data is plotted for all bonds involving headgroup (HG), phosphate (PO4), glycerol moieties (GL1, GL2) and the palmitoyl tail of the lipids for the asymmetric (upper panel) and IV symmetric (lower panel) bilayers.

To ascertain that the self-assembly simulations indeed led to proper bilayer structures, we first analyzed self-assembly simulations without cholesterol. The stability analysis was done based on visual inspection, and evolution of the system’s potential energy (Fig 3A) and of box dimension along x-direction (Fig 3B), which showed that the system is well equilibrated within 50 ns. The density profiles of different lipid moieties and water relative to membrane center along the membrane normal show the expected distributions (Fig 3C).

For independent comparison, two symmetric bilayers were also generated based on the upper (IV symmetric) or lower leaflet (CP symmetric) compositions as resulting from self-assembly of the asymmetric membrane using the INSANE method (INSert membrANE) CG building tool [28] and equilibrated for 500 ns. The calculated APL values for the self-assembled bilayer are 0.66 nm^2^ (IV) and 0.64 nm^2^ (CP), considering number of lipids in either leaflets separately. The IV symmetric bilayer has an APL of 0.66 nm^2^ while the CP symmetric bilayer, highly enriched in PE and PS lipids, shows an APL of 0.64 nm^2^. For further comparison, the second-rank lipid order parameter was also calculated for the different systems according to S_2_ = $\langle \frac{3{cos}^{2}\theta -1}{2}\rangle$, where *θ* is angle between the bond vector of two consecutive beads and the bilayer normal. Fig 3D shows the lipid order parameters of phospholipids in the asymmetric and the IV symmetric bilayers. The lipid order parameter for the phospholipids in the CP symmetric membrane (not shown) was identical to that in IV symmetric bilayer. The good agreement between the properties of asymmetric (Table 3) and symmetric bilayers indicates that the self-assembly simulations lead to correct bilayer structures.

**Table 3:** Characteristics of asymmetric membrane systems.

Asymmetric Membrane	Membrane Thickness (nm)	APL (nm^2^)		Lipid order parameter ^a^	Lateral diffusion coefficient (cm^2^ s^-1^)^b^	
		IV	CP		IV	CP
Planar without cholesterol	4.27 ± 0.02	0.66 ± 0.00	0.64 ± 0.00	0.354	6.81 ± 0.0	6.24 ± 0.3
Planar	4.46 ± 0.02	0.44 ± 0.01^c^	0.44 ± 0.01 ^c^	0.512	2.50 ± 0.0	2.49 ± 0.3
Vesicle	4.38 ± 0.03	0.38 ± 0.01 ^c^	0.51 ± 0.01 ^c^	0.510	1.80 ± 0.7	2.96 ± 0.7

^a^ Average second-rank lipid order parameter for PC, PE and PS lipids.

^b^ Calculated for phospholipids using g_msd utility included in GROMACS.

^c^ Calculated using Voronoi tessellation method.

**Fig 4:**
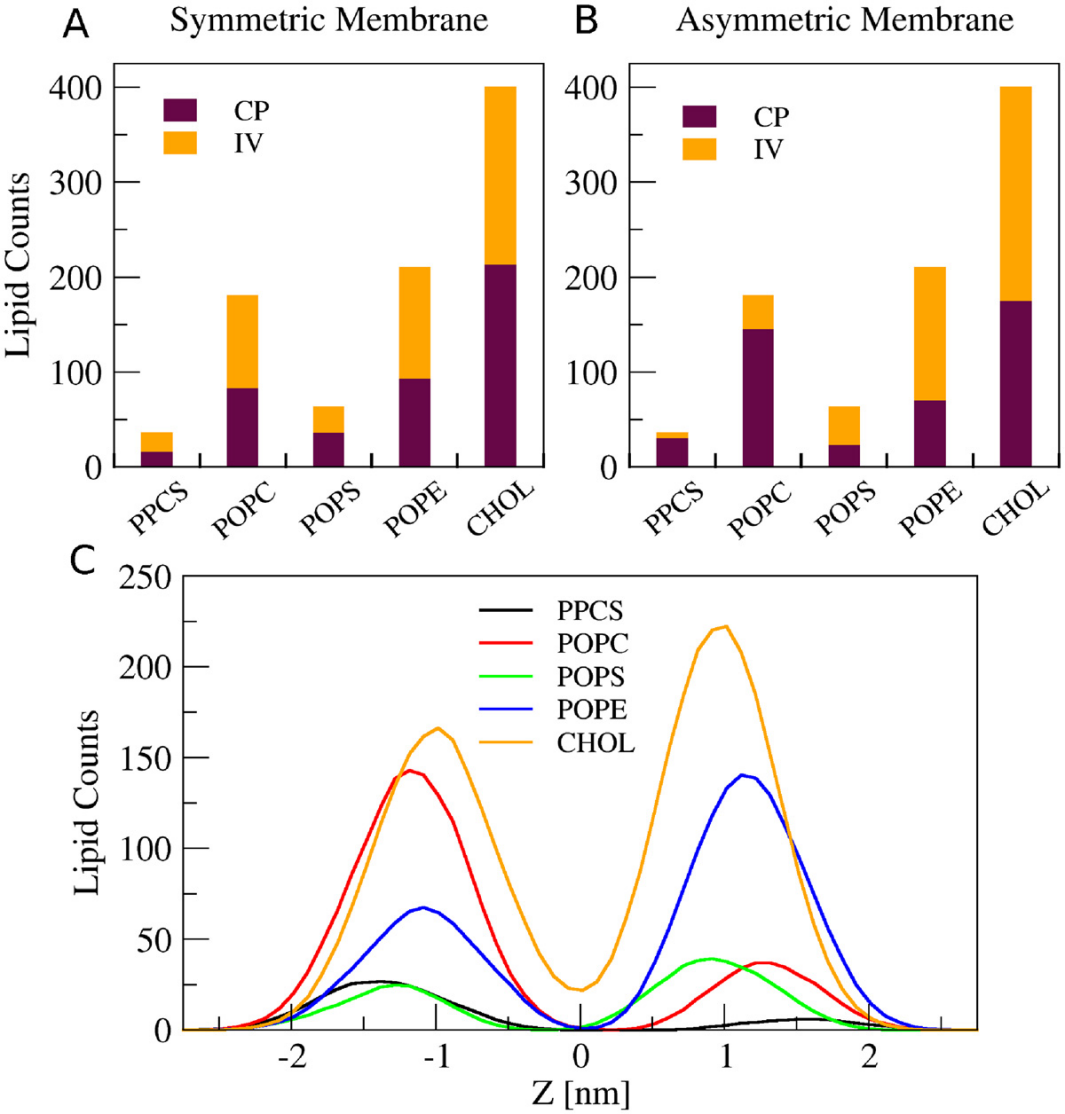
Lipid compositions in symmetric and asymmetric membrane. (A) The membrane resulting from self-assembly of randomly mixed lipids exhibited a symmetric distribution for every type of phospholipid and cholesterol. (B) In membrane membranes resulting from asymmetric initial configuration, all lipid types were asymmetrically distributed. As in physiological membranes, PPCS and POPC were most abundant in the IV leaflet and POPS and POPE most abundant in the CP leaflet. Cholesterol was symmetrically distributed. (C) Frequency histograms of the distance of lipids’ center of mass to membrane’s center of mass (z = 0) shows the asymmetric lipid composition and also shows the presence of a small fraction of cholesterol located at the membrane center.

Next, we analyzed the properties of asymmetric membranes in the presence of cholesterol and compared them to those of self-assembled symmetric bilayers. Fig 4 shows a comparison of lipid compositions in the two leaflets of membranes formed by self-assembly starting from either symmetric (Fig 4A) or asymmetric (Fig 4B) lipid distributions at the end of 200 ns simulations. Starting from symmetric lipid distributions, the lipid head group count revealed a symmetric distribution of all types of lipids and cholesterol between the two leaflets (Table 2, under “Symmetric at 200ns”), as expected. For asymmetric starting conditions, PPCS and POPC were mostly found in the IV leaflet and POPS and POPE were mostly found in the CP leaflet (Table 2, Under “Asymmetric at 200ns”). Cholesterol was evenly distributed between CP and IV leaflets as described previously. The leaflet asymmetry is also evident from analysis of the lipid head group densities averaged over the last 50 ns of simulation time in the asymmetric membrane (Fig 4C). The leaflet asymmetry of the different lipids, was unchanged when cholesterol was omitted in the simulation (Table 2, “Without cholesterol at 200ns”).

The bilayer thickness and APL were not significantly different between symmetric and asymmetric membranes of the same overall composition, with a thickness of 4.5 nm and APL of 0.8 nm^2^. However, these values were markedly different from those of DPPC membranes, which had 4.0 nm thickness and 0.64 nm^2^ APL. The difference in APL is entirely due to the presence of cholesterol as is evident from results obtained with SV lipids in the absence of cholesterol, with an APL of 0.65 nm^2^. In contrast, the bilayer thickness without cholesterol (4.3 nm) was intermediate between that of the DPPC membrane (4.0 nm) and that of the SV lipid membrane including cholesterol (4.5 nm) (Table 3), indicating that cholesterol produces a moderate increase in membrane thickness. Further, the ordering effect induced by the presence of cholesterol in membranes is also evident from a higher value of the lipid order parameter compared to cholesterol free membrane (Table 3). Next, we calculated the lateral diffusion coefficient of phospholipids from last 500 ns of one of the representative simulations that was extended to 1**μ**s. Lipid diffusion was calculated using the built-in g_msd utility of GROMACS. The lateral diffusion coefficient in the asymmetric membrane with cholesterol was ~2.5 x 10^−7^ cm^2^ s^-1^. This is in excellent agreement with a previous observation of 2.6 x 10^−7^ cm^2^ s^-1^ [32]. The lipid diffusion coefficient in absence of cholesterol is roughly 3-fold higher.

### Self-assembly of asymmetric vesicle membrane

**Fig 5:**
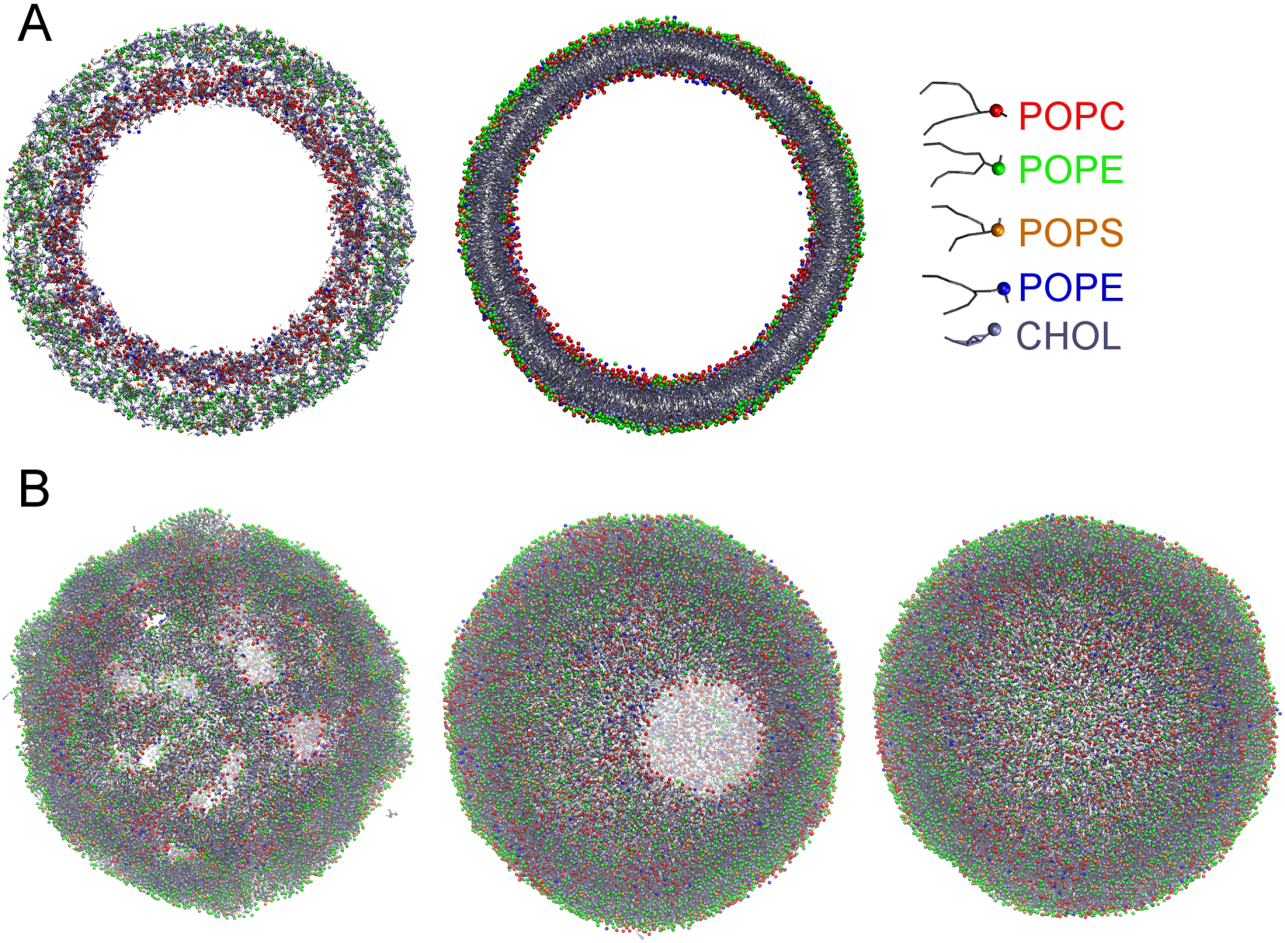
Self-assembly of an asymmetric vesicle. (A) A cut-away view of the vesicle at the start (0 ns) and at the end (135 ns) of the self-assembly simulation. The representation and coloring scheme is also shown. The head groups of different lipids are shown as spheres, the tails as sticks. (B) Snapshots of the entire vesicle illustrating the dynamics of the pores at different simulation times. Several pores formed within a few nanoseconds (2 ns). These pores coalesce to form a larger pore (25 ns) and finally the vesicle is sealed (60 ns).

Synaptic vesicles are highly curved with a typical mean diameter of 35–40 nm [33]. For self-assembly of such a vesicle, simulations were started from a configuration with two concentric shells (Fig 5A, left). The inner shell containing 720 PPCS, 3600 POPC and 2880 CHOL molecules (IV lipids) had inner and outer diameters of 15 nm and 20 nm, respectively. The outer shell containing 1260 POPS, 4300 POPE and 5120 CHOL molecules (CP lipids) had an inner diameter of 19.5 nm, overlapping 0.5 nm with the inner shell, and an outer diameter of 24.5 nm, like that of the SVs found near the low end of SV size distributions [33]. From this starting configuration an approximately spherical bilayer vesicle was formed (Fig 5A, right). Fig 4B shows snapshots at various time points during the simulation. Within a few nanoseconds, the lipids in the two shells self-assembled into a bilayer with several water pores. These pores facilitate lipid exchange between the inner and outer shells. As illustrated in Fig 5B, the pores coalesced quickly to form a larger pore, allowing further equilibration of water across the forming vesicle. Within 60 ns, the pore was sealed and a spherical vesicle was formed with no visually detectable pores. The transient formation of such water pores during the initial stages of vesicle formation has also been observed in previous studies on self-assembly of vesicles with symmetric leaflet composition in both atomistic and coarse-grained models [34, 35].

**Fig 6:**
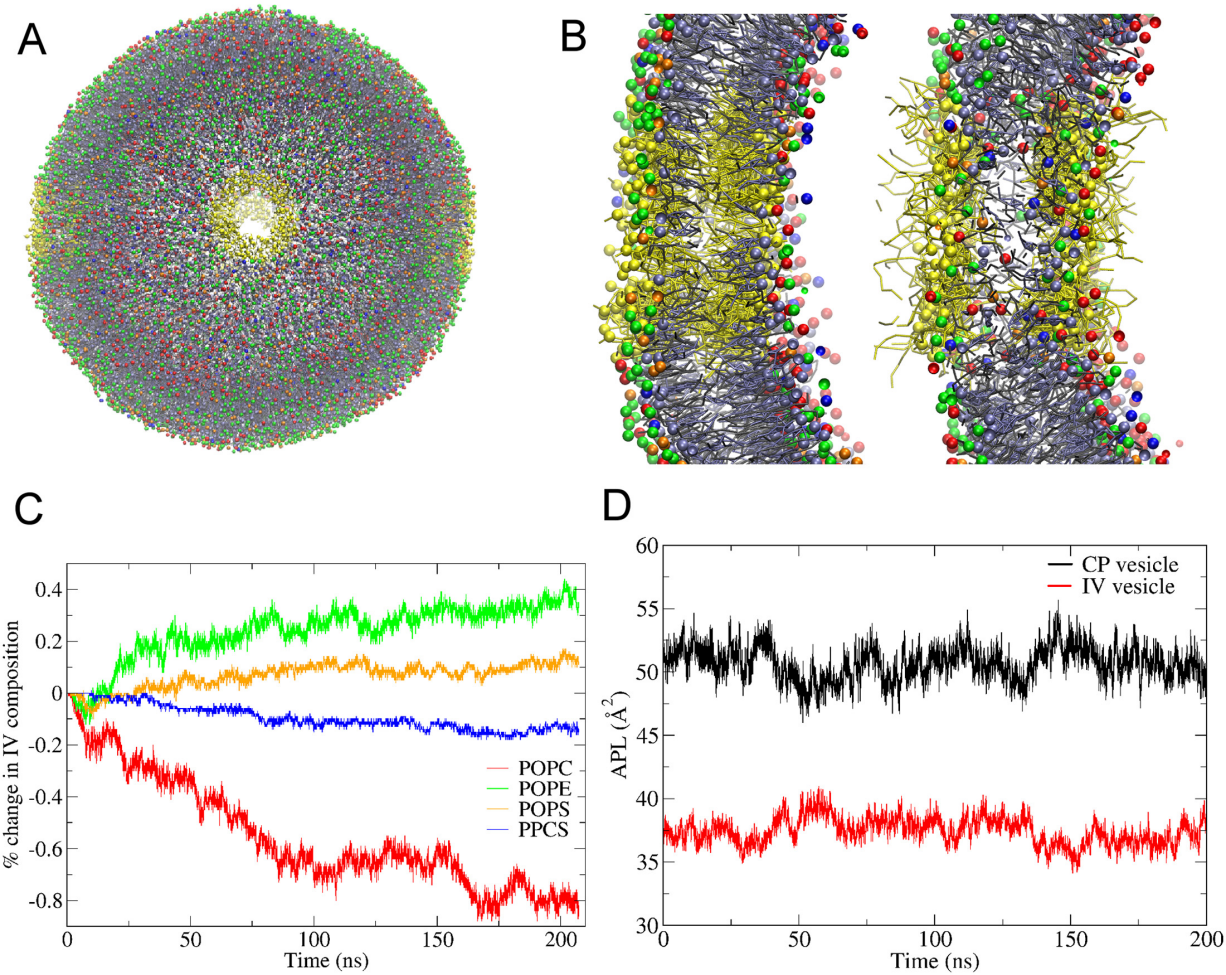
Equilibration in presence of “water-lined” pores. (A) Vesicle showing the four “water-lined” pores (front, back, left, right) that were introduced into the self-assembled vesicle membrane to allow equilibration of lipid densities. The representation of vesicle lipids is same as in Fig 4, except for the wlipids (lipids with water particle type, yellow). (B) Cross section of one of the pores at the start (left) and the end (right) of a 200 ns equilibration. Note the tails of the wlipids (yellow) occupy the interior of the membrane at the start whereas they point away from the bilayer into the bulk solvent at the end. Also note the head groups of lipids that can be seen in the membrane interior, absent in left panel, showing the lipid exchange between the two leaflets. (C) The percent change in lipid composition of the IV leaflet of the vesicle during the equilibration. (D) Time evolution of APL of the CP and IV leaflets measured using Voronoi analysis on a patch of bilayer taken from the equilibrating vesicle.

To determine if the densities of lipids in the inner and outer leaflets are at equilibrium, artificial ”water-lined pores” were introduced into the vesicle membrane. A vesicle with four such “water-lined pores” is shown in Fig 6A and a detailed view of one such pore in Fig 6B. These pores were generated in the vesicle bilayer by removing all the lipids within a transmembrane cylinder of 1.5nm radius, with axis of the cylinder lying along +x, -x, +y and–y directions from the center of mass of the vesicle. Then, for each pore the particle types of all the lipids that lay within a shell of 1.7 nm from the pore surface were changed to a water particle (P4). All the bonded interactions of these modified lipids (referred herein as “wlipids”) were kept unchanged. To prevent the collapse of the pores due to diffusion of wlipids into bulk water, all the particles corresponding to the phosphate headgroups (or hydroxyl group of cholesterol) in the wlipid molecule were position restrained using a force constant of 300 kJ mol^-1^ nm^-1^. The vesicle was then subjected to a 200 ns equilibration simulation. During the equilibration run, the wlipid tails moved away from the interior of the bilayer, pointing into the surrounding water region, as expected (compare left and right panels of Fig 6B). The pores remained open throughout the equilibration by using position restraints on one single particle per wlipid. Introducing such pores provides a hydrophilic surface across the membrane that allows rapid equilibration of lipid densities across the vesicle bilayer. The extent of lipid exchange between the two monolayers was assessed by following the change in number of the individual lipid types in the inner monolayer (IV). Fig 5C shows rather low rates of lipid flip-flop between the two monolayers during the course of 200 ns long simulations. To determine if the lipid densities in the inner and outer leaflet were at equilibrium, we calculated the molecular APL in IV and CP leaflets using the Voronoi tessellation method [36] on patches of lipid bilayer excised from the vesicle membrane. The time-dependent APL evolution during the equilibration in the presence of the”water-lined pores” (Fig 6D) shows no net change, indicating that the two leaflets were well equilibrated with an APL of 37.5 ± 1.1 Å^2^ for IV leaflet and 50.7 ± 1.4 Å^2^ for CP leaflet. For comparison, we also calculated the APL for the self-assembled asymmetric planar bilayer (see previous section) using same procedure, giving 44.4 ± 1.3 Å^2^ and 44.1 ± 1.1 Å^2^ for IV and CP leaflets, respectively. It is interesting to note that, compared to the APL for the planar bilayer, the APL for the inner leaflet is much smaller while that for the outer leaflet is much larger but the mean is identical with the planar bilayer APL.

### Characteristics of asymmetric vesicle membrane

**Fig 7:**
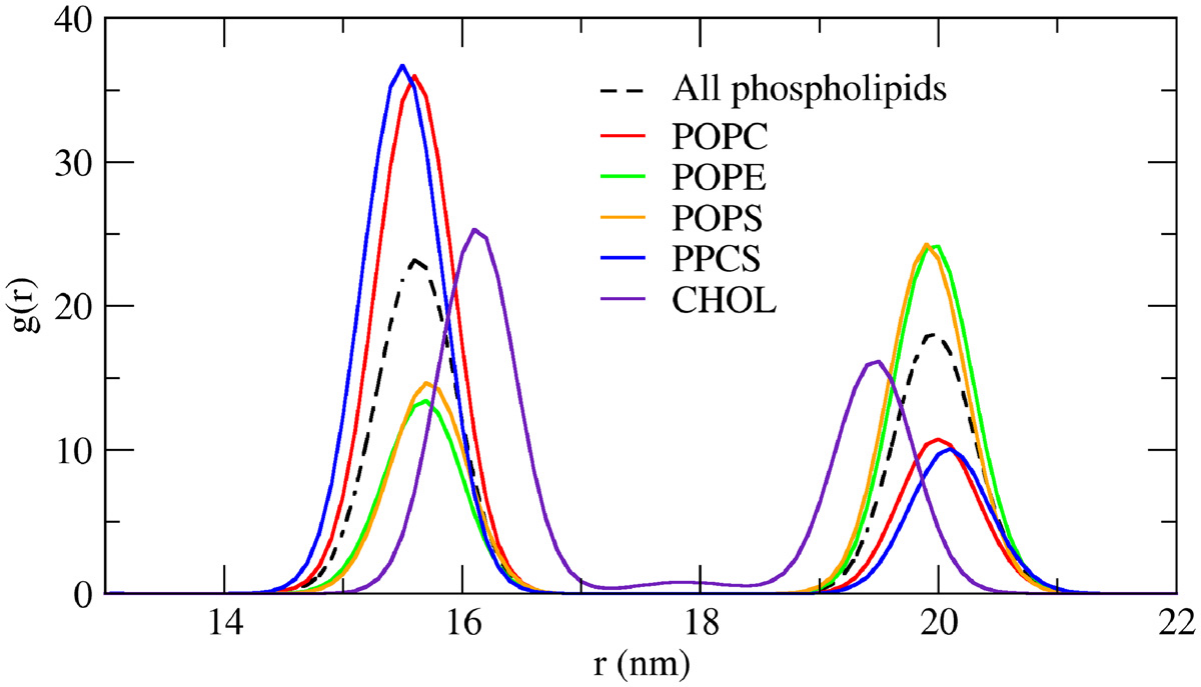
Lipid distribution in an equilibrated vesicle. Radial distribution function (RDF) g(r) for the phosphate beads of different lipid types as a function of distance from the center of mass of the entire vesicle. The vesicle size is calculated by following the inner and outer peaks in g(r) for the phosphate beads for all the phospholipids taken together (dashed line).

After the 200 ns equilibration of vesicle in the presence of “water-lined pores”, and ascertaining the convergence of lipid densities and APL values, the vesicle was subjected to another round of free equilibration. For the free equilibration, the wlipids were reverted back to the corresponding original lipid types and no position restraints were applied. The composition of the vesicle membrane leaflets is listed in Table 4. The vesicle showed an appreciable degree of asymmetry in its lipid composition. As can be deduced from the table, the CP leaflet is enriched in POPS (0.73) and POPE (0.75) lipids, with lesser amounts of PPCS (0.32) and POPC (0.34). Similar to the asymmetric bilayer, we find a near equal distribution of cholesterol in the two leaflets. The radial distribution functions g(r) (Fig 7) of phospholipid and cholesterol head groups (PO4 and ROH particles) were determined from last 50 ns of the free equilibration trajectory, using a bin width of 0.1 nm. The distributions show PO4 head group peaks at 15.6 nm and 20.0 nm, indicating a vesicle bilayer thickness of 4.3 nm with inner and outer radii of 15.6 nm and 20 nm. Considering these radii, the inner and outer surface area amounts to 3058 nm^2^ and 5027 nm^2^. The total number of lipids in the inner (IV) and outer (CP) leaflets are 8231 and 9361, as calculated from Table 4. From these, the calculated inner and outer APL are 37.2 Å^2^ and 53.7 Å^2^, which are in close agreement with the APL calculated above using the Voronoi procedure.

**Table 4:** Comparison of lipid number and ratios in vesicle and bilayer.

Lipids	Vesicle			Planar Bilayer		
	Total number (fraction) ^a^	IV leaflet number (fraction) ^b^	CP leaflet number (fraction) ^b^	Total number (fraction) ^a^	IV leaflet number (fraction) ^b^	CP leaflet number (fraction) ^b^
PSM(PPCS)	712 (4.0)	487 (5.9)	225 (2.4)	36 (4.0)	3 (6.8)	6 (1.3)
PC(POPC)	3561 (20.2)	2368 (28.8)	1193 (12.7)	180 (20.2)	145 (32.7)	35 (7.8)
PS(POPS)	1244 (7.1)	336 (4.1)	908 (9.7)	63 (7.1)	23 (5.2)	40 (9.0)
PE(POPE)	4163 (23.7)	1034 (12.6)	3129 (33.4)	210 (23.6)	70 (15.8)	140 (31.4)
CHOL	7912 (45.0)	4006 (48.7)	3906 (41.7)	400 (45.0)	175 (39.5)	225 (50.4)

^a,b^ Fractions are in percentage of each lipid type over the total number of lipids in the membrane ^a^ or in the respective leaflets ^b^.

The structural and dynamical characteristics of the lipids in the asymmetric vesicle are summarized in Table 3 (last row). The lipid order parameters were calculated from the membrane patches used for the Voronoi tessellation, as described above (also see Materials and Methods). To be statistically rigorous the equilibration was extended to 500 ns and the lipid order parameters were calculated from the (approximately planar) membrane patches normal to x-, y- and z- axis and were then averaged. The lipid order parameters was found to be very similar to that observed for the planar asymmetric bilayer (Table 3). Further, we also calculated the lipid diffusion coefficient of the lipids in these membrane patches over 20 ns intervals extending over 300 ns. The diffusion of lipids in CP leaflet (2.96 x 10^−7^ cm^2^ s^-1^) is higher than that of lipids in the inner IV leaflet (1.80 x 10^−7^ cm^2^ s^-1^), presumably due to denser packing of lipids in the IV monolayer, Similar difference of lipid diffusion between the two leaflets has previously been reported before for a DPPC vesicle [37].

### Membrane protein insertion during self-assembly of asymmetric membranes

Self-assembly of membranes is particularly useful to determine the positioning of proteins in the membrane. Here we investigated two examples, the insertion of the plasma membrane protein syntaxin-1A (Stx1A) during self-assembly of a planar membrane and the insertion of the most abundant synaptic vesicle protein synaptobrevin 2 (Syb2, also known as VAMP2) [6]. The CG structures of the protein models were based on the crystal structure of the SNARE complex 3HD7 [38]. For the simulations of Stx1A insertion, a C terminal fragment consisting of the transmembrane domain and the juxtamembrane domain with a total length of 48 residues was used, while Syb2 insertion was simulated using the full structure of residues 26 to 116.

#### Insertion of syntaxin in planar asymmetric membrane

**Fig 8:**
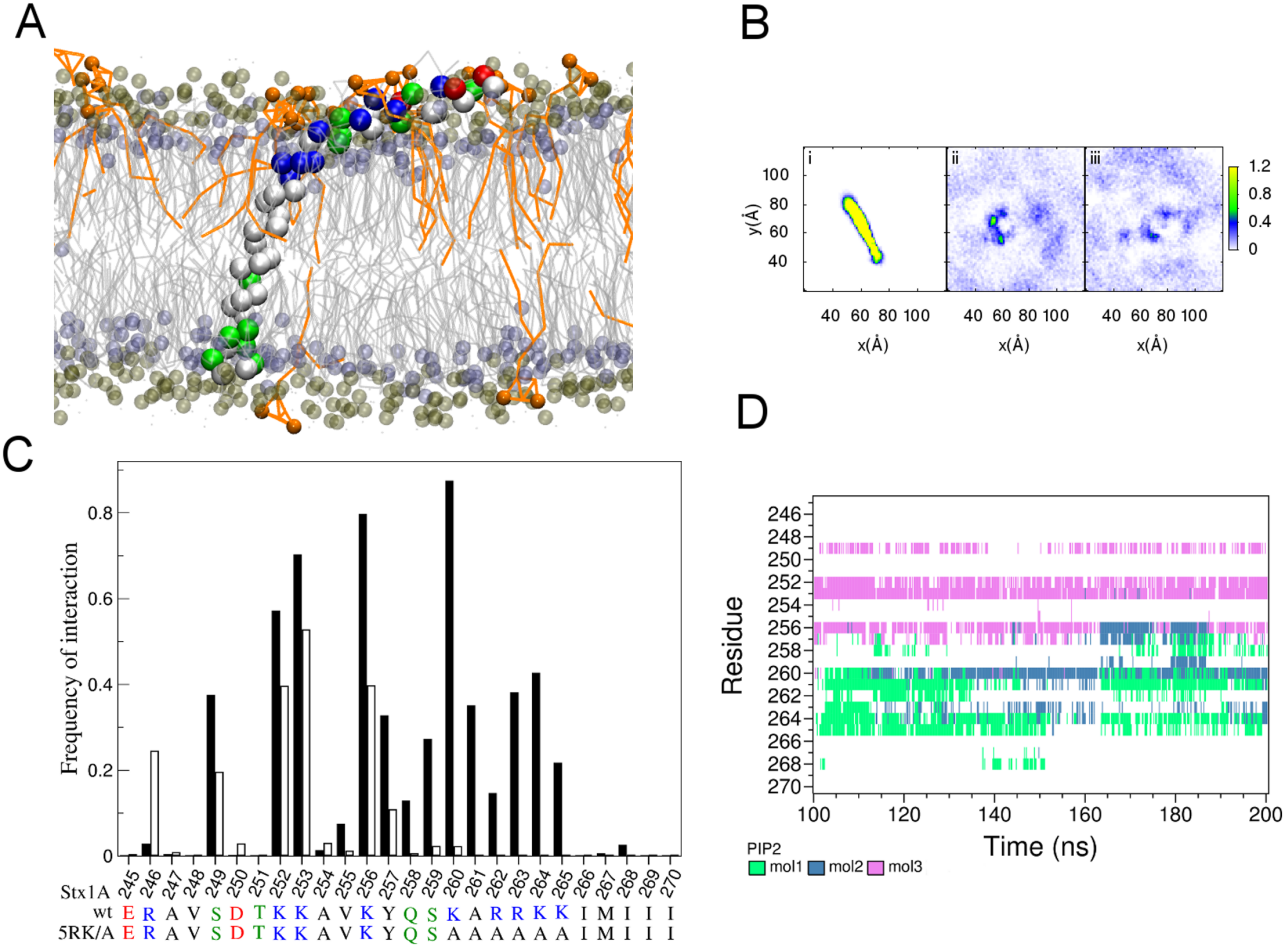
Self-assembly of stx1A in an asymmetric bilayer. (A) Snapshot of the insertion of stx1A in in the self- assembled bilayer (200 ns). The protein is shown in vdw representation and colored according to residue type. The lipids are shown in grey with phosphate group of phospholipids colored in tan and headgroup of cholesterol in purple. The PIP2 molecules are shown in orange. (B) Alignment of Stx1A (i) and surrounding PIP2 densities for Stx1A wt (ii) and 5RK/A mutant (iii) calculated from last 100 ns of three independent simulations. (C) Relative frequency of PIP2 interactions with residues 245 to 270 from the last 100 ns of 3 independent 200 ns simulations of Stx1A wt (filled bars) and 5RK/A mutant (open bars) (D) Trajectories of interaction of 3 individual PIP2 molecules (mol1 to mol3) with residues 245–270 of stx1A from one of the simulations.

Stx1A is a plasma membrane protein that shows strong interactions with the plasma membrane lipid phosphatidylinositol-bisphosphate (PIP2) [39–41]. Therefore, PIP2 was included in the CP lipid box at 3% concentration replacing the same number of POPC molecules. The extracellular (EC) lipids were identical to the IV lipids as described above. After self-assembly the CP leaflet composition was 7 PPCS 28 POPC 16 PIP2 41 POPS 108 POPE and 172 CHOL. Fig 7A shows the final snapshot of the self-assembled membrane with the transmembrane domain completely embedded in the membrane. The C-terminal end of Stx1A was found to be anchored to the EC leaflet and the linker and the SNARE motif interacting with the CP leaflet. We also see a few PIP2 molecules (Fig 8A, orange) interacting with basic residues present in the Stx1A linker domain, as observed previously in atomistic simulations of Stx1B [41].

To visualize the specific localization of PIP2, all simulations were aligned relative to the Stx1A peptide (Fig 8Bi) and the PIP2 densities averaged over the last 100 ns of three independent simulation trajectories. Fig 8Bii reveals a marked concentration in the PIP2 density in the CP leaflet around the Stx1A transmembrane domain. The positively charged stretch K260/A261/R262/R263/K264/K265 (5RK) is required for binding of acidic lipids since it was shown that the K260A/R262A/R263A/K264A/K265A (5RK/A) mutant does not bind acidic lipids [40]. To show the specificity of interactions between PIP2 and Stx1A in the simulations, we compared the PIP2 clustering around wild type Stx1A with that around the Stx1A 5RK/A mutant, which showed markedly reduced PIP2 densities near the transmembrane domain (Fig 8Biii). For a more quantitative analysis we determined the relative frequencies of PIP2 interactions with the individual residues 245 to 270 (Fig 8C). Stx1A wt (Fig 8C, filled bars) shows strong PIP2 interactions with the positively charged 5RK region, but these interactions extend to K256, in excellent agreement with a recent all atom simulation study [41]. We also see frequent PIP2 interactions with K252/K253 as well as S249, which were not previously reported. In the 5RK/A mutant (Fig 8C, open bars) the PIP2 interactions with residues 258–265 are completely lost, demonstrating the specificity of the observed PIP2 interactions with these basic residues. Fig 8D illustrates the time course of the interactions of individual PIP2 molecules with the individual Stx1A residues. In this example PIP2 “mol1” interacts mainly with the 5RK stretch while “mol 2” interacts mostly with K260 but also K256 and R263/K264. PIP2 “mol3” interacts with K252/K253/K256 and this interaction extends somewhat to S249.

#### Insertion of synaptobrevin in asymmetric vesicle membrane

**Fig 9:**
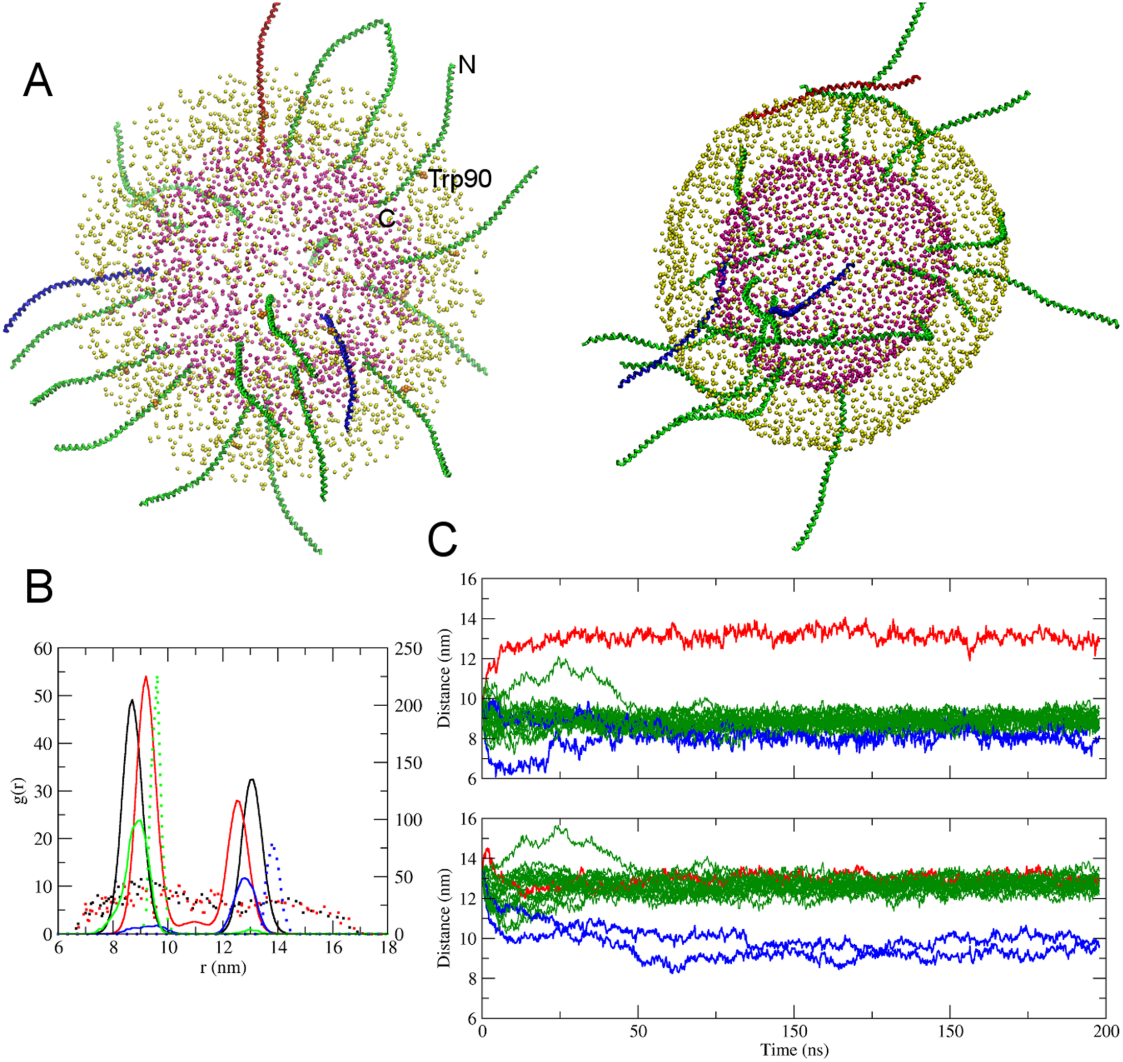
Self-assembly of the vesicle-protein system. (A) Snapshot of vesicle at the start (left) and at the end (right) of self-assembly done along with twenty copies of syb2 molecules. To aid visualization, only the phosphate headgroups of the lipids and the backbone beads of the protein are shown. The phosphate headgroups of the outer leaflet are shown in yellow and the inner leaflet in magenta. The backbone beads of Syb2 copies that positioned themselves correctly are shown in green; three syb2 molecules that failed to position themselves correctly are colored red and blue. To illustrate the initial positioning of syb2, the left panel also shows theTrp90 in vdw representation and colored orange. The N- and C-term is also labeled for one of the syb2 molecule. (B) Comparison of the RDF g(r) of the lipid phosphate groups (black), cholesterol (red), Thr116 (green) and Trp90 (blue) at the start of the simulation (dotted) and during the last 50 ns of the self-assembly (solid). Note the small RDF peak of Thr116 (green solid) near the outer leaflet phosphate headgroup (red molecule in A) and the appearance of a Trp90 peak (blue solid) near inner leaflet phosphates (blue molecules in A), originating from misoriented syb2 molecules. (C) Evolution of the distance of backbone beads of Thr116 (top) and Trp90 (bottom) residues of all twenty protein molecules from the center of mass of the forming vesicle. The traces are colored following the coloring scheme of the molecules as shown in A.

To demonstrate insertion of a membrane protein during self-assembly a vesicle with asymmetric lipid composition we chose the ~13.5 nm long fragment of the N terminal residues 26 to 116 of Syb2 as available in the crystal structure 3HD7. The SNARE motifs of Syb2 are located on the cytoplasmic side of the synaptic vesicle membrane [42] and were thus oriented toward the CP lipids in the initial set-up while the C-terminus was oriented toward the IV lipids. To facilitate natural positioning of Syb2 in the self-assembling membrane, the entire transmembrane domain (W90 to T116) was completely embedded in the inner shell of lipids (Fig 9A, left panel). The final state after a 197 ns self-assembly simulation including 20 Syb2 copies is shown in Fig 9A (right panel). Analysis of the radial distribution functions of the phospholipid headgroups over the last 50 ns of the simulation (Fig 9B, black) indicates formation of a vesicle with an outer radius of 13.1 nm and a membrane thickness of 4.4 nm, indistinguishable from that of the protein-free vesicle. The C terminal residues of Syb2 (T116, Fig 9B, green) localized to the inner leaflet head groups except for one copy of Syb2 that failed to insert and ended up on the cytoplasmic surface of the vesicle, (Fig 9A, red). The movement of T116 of this Syb2 copy to the outside occurred during the first 10 ns of the simulation (Fig 9C, red traces) Residue W90 was localized just inside the outer leaflet head group region (Fig 9B, blue) as expected [43] except for two copies of Syb2 which inserted incorrectly with the transmembrane domain lying on the intravesicular membrane surface and residues 60–90 (SELDDRADAL QAGASQFETS AAKLKRKYWW) in transmembrane position. The time course of ~50 ns to achieve this position (Fg. 9C, blue traces) corresponds to the time needed to form the final bilayer membrane. The remaining 17 copies of Syb2 (Fig 9C, green traces) had the transmembrane domain correctly positioned and oriented.

## Discussion

We present, to our knowledge, the first CG MD method to generate membrane model by self-assembly that closely resembles cellular membranes by adapting not only the lipid composition but also leaflet asymmetry. For self-assembly of a planar membrane two simulation boxes filled with different lipids were created for CP and IV leaflets, respectively, and stacked on top of each other with 0.5 nm overlap to eliminate excess water insertion in between CP and IV boxes. The 0.5 nm overlap prevented formation of two separate membranes and reproducibly led to self-assembly of a membrane with asymmetric lipid composition. The self-assembled membranes showed a leaflet asymmetry very close to that of mammalian cell membranes. It was reported that cholesterol may also be asymmetrically distributed in cell membranes. Simulations starting with asymmetric distribution of cholesterol in CP and IV lipid boxes did, however, not result in a cholesterol asymmetry, due to the rapid flip-flop rate of cholesterol. The asymmetric cholesterol distribution in cell membranes must therefore be maintained by an active mechanism. The asymmetric membrane was 0.5 nm (12.5%) thicker than the, widely used, 4.0 nm DPPC membrane, which was caused by both presence of cholesterol and the nature of the various phospholipids used here. The APL in the asymmetric membrane was 0.16 nm^2^ (25%) larger than the 0.64 nm^2^ APL in a DPPC membrane, which was almost entirely caused by the presence of cholesterol.

A similar approach was developed to achieve self-assembly of a vesicle membrane. To form a vesicle with asymmetric lipid distribution between inner and outer leaflets, the different lipids were arranged in two different concentric shells, which were then also placed with 0.5 nm overlap. The self-assembly from this starting configuration produced a spherical vesicle. We also demonstrate here the use of a novel approach of “water lined pores” for equilibration of water and lipid densities across the vesicles. The equilibrated vesicle was found to be asymmetric with higher densities of PE and PS lipids in the outer leaflet (CP leaflet) as compared to their densities in the inner leaflet. On the other hand, the inner leaflet showed a higher density distribution of PC and PSM lipids, similar to the self-assembled planar bilayer. The APL estimation showed that the APL of inner layer of the vesicle was smaller and APL of the outer layer was larger than the mean APL for the whole vesicle (44.0 A2), which was equal to the mean APL for the planar bilayer. The lipid densities of inner and outer leaflet are thus equal in the membrane center but decrease from the inner headgroup shell to the outer head group shell due to the radial area expansion.

Routinely used methods to study protein interactions with specific lipids such as PIP2 in MD simulations involve use of a pre-formed lipid bilayer into which the protein is embedded and where specific lipids are randomly distributed in both leaflets [44] or placed at ad-hoc chosen positions [41, 45]. In the approach described here, the simulation starts from an asymmetrically distributed otherwise random mixture of lipids and during self-assembly both the protein and lipids are free to localize to regions of an energy minimum. The self-assembly of membranes in CG MD simulations is particularly useful as an unbiased method to characterize insertion and positioning of membrane proteins and as shown here reveals the interactions of PIP2 with syntaxin-1A consistent with experimental data and atomistic simulations.

In the example presented here, we illustrate the method to characterize insertion of Stx1A in the membrane and its interactions with PIP2. The simulation was started from a random mixture of CP and IV leaflet lipids containing 3 mol% of PIP2 in the CP lipid mixture. It is noteworthy that the CG self-assembly method, reproduces the PIP2 interactions with a charged stretch including K256/K260/R262/R263/K264/K265 previously observed in all atom simulations [41] in spite of the limited handling of electrostatic interactions in the MARTINI force field. The interactions are abolished in the 5RK mutant that lacks the charged stretch and considerably reduces PIP2 interactions [39, 46]. In addition, we observed PIP2 interactions with S249/K252/K253/K256, which were not observed in the previous study of full length Stx1B [41]. The reason for this difference is that in [41] the linker region was pointing away from the membrane, whereas in our simulations the linker and the included part of the SNARE domain were attaching to the membrane surface. This attachment of the Stx1A SNARE domain to the membrane surface was also observed when a larger Stx1A fragment was used including the whole SNARE domain starting at residue 189 (data not shown) and is presumably facilitated by interactions of the hydrophobic face of the SNARE domain with the membrane surface. In the closed state stricter used in [41], this hydrophobic face is shielded by the Habc domain, which points to a possible significance of the Habc domain to prevent binding of the Stx SNARE domain to the membrane.

We also demonstrate the feasibility of this approach to insert proteins in a vesicle membrane. We successfully inserted multiple copies of a flexible synaptic vesicle protein syb2, in an asymmetric vesicle using the self-assembly method. Starting from asymmetrically placed lipids in two overlapping concentric shells of randomly distributed IV and CP lipids, respectively, along with twenty copies of syb2, the self-assembly resulted in an asymmetric vesicle with 17 of the 20 copies of syb2 positioned such that the TM domain spanned the vesicle bilayer. In these 17 molecules, Thr116 was anchored to the head groups of IV leaflet and Trp90 was positioned below the head group region of CP leaflet. The method thus properly reveals the energetically preferred physiological positioning of the TM domain in the membrane.

In summary, we have demonstrated the formation of bilayer membranes in CG MD simulations employing the Martini force field, which have physiological lipid composition and leaflet asymmetry. In contrast to existing methods of asymmetric membrane simulations [26–28], the approach presented here does not depend upon *a priori* assumptions such as the APL or number of lipids in the different leaflets and leads to rapid asymmetric bilayer formation by self-assembly. CG MD self-assembly simulations are an important tool to study membrane protein insertion [29] but were restricted to bilayers with *symmetric* lipid composition. Here we extended this approach to study membrane protein insertion as well as specific lipid-protein interactions in bilayers with asymmetric lipid composition. The approach will enable more realistic simulations of membrane proteins in membranes with physiological properties. The nature and properties of multicomponent asymmetric bilayers can also be studied in atomistic detail [24, 47–53]. To generate atomistic models for such studies, the CG self-assembly of asymmetric bilayers including membrane proteins as described here can provide models that can be converted into atomistic representations for refined atomistic simulations [44, 54].

## Materials and Methods

All the simulations were conducted using ver. 4.5.4 GROMACS (GROningen Machine for Chemical Simulations) [55]. Lipid models used in the simulations, POPS, POPE, POPC, PPCS, and DPPC, and the cholesterol model were CG Martini models and simulated using Martini force field ver. 2.0 [56]. Structures of Syb2 (residues 26–115) and Stx1A (residues 241–286) including the transmembrane domains were obtained from the X-ray crystallography structure [38] as a PDB (Protein Data Bank) file. The missing C-terminal residues of Syb2 (residue 116) and Stx1A (residues 287–288) were added using Modeller [57]. The atomistic structures were then coarse-grained using a PerlScript file adapting Martini coarse-graining method [56].

The starting configurations for the bilayer was constructed by combining two separately generated CP and IV lipids box of 16 nm × 16 nm × 5 nm each. The CP lipids box was generated by randomly placing 63 POPS, 210 POPE and 256 CHOL molecules. The IV lipids box was generated by randomly placing 36 PPCS, 180 POPC and 144 CHOL molecules. The two boxes were then merged into one box of dimension 16 nm × 16 nm × 13 nm after shifting the center of mass of the CP lipids box by 5 nm (no overlap) or by 4.5 nm (0.5 nm overlap). The resulting lipids box was then filled with CG water and appropriate number of Na+ ions were added to preserve electro-neutrality. This was followed by 1000 steps of energy minimization using steepest descent algorithm after which a production run was carried out for 200 ns. For membrane-protein simulations, CP and IV boxes were generated the same way as above, except that a 3% mol of PIP2 was added by removing same number of POPC lipids. Additionally, Stx1A protein was inserted. Prior to its insertion, the protein was positioned at the center of a box. The protein was oriented so that C-terminus would be surrounded by IV lipids when they are combined, and was combined with CP and IV boxes to produce SV lipids box with Stx1A protein inserted. The resulted SV lipids box with Stx1A was filled with water and Na+ ions and energy minimized with the same method described above.

The vesicle self-assembly was carried out in three successive steps:

1. Spontaneous aggregation—The starting system for the vesicle simulation was assembled from two spherical shells of CP and IV lipids, with lipids randomly seeded in each shell using Packmol software [58]. The CP lipids shell consisted of 1260 POPS, 4300 POPE and 5120 CHOL molecules. The IV lipids shell consisted of 720 PPCS, 3600 POPC and 2880 CHOL molecules. The inner and outer diameters of IV lipids shell were 15 nm and 20 nm. The inner and outer diameters of CP lipids shell were 19.5 nm and 24.5 nm, allowing an overlap of 0.5 nm. The resulting system was then placed in a cubic box, 53.0 nm on each side. The box was then filled with CG water particles and appropriate number of Na^+^ ions were added to preserve electro-neutrality. The system was then subjected to three rounds of 1000 steps of steepest descent energy minimization, 200 ps of equilibration using a time step of 2 fs and 100 ps of equilibration using a time step of 10 fs. Following this, a 135 ns long simulation was carried out with an integrating time step of 20 fs.
2. Equilibration with”water-lined pores”—To ensure the equilibration of lipids between the inner and outer monolayers and of water in the interior of vesicle, four”water-lined pores” were formed. Each pore was generated by removing all the lipids in a cylindrical shell of radius of 1.5 nm along the ±x- and ±y-axis. Further, the atom types of all the lipids within a shell of 1.7 nm around each pore surface were changed to that of water (P4). The modified lipids are referred to as “wlipids”. To prevent the collapse of the pores, the atoms corresponding to PO4 or ROH of the wlipids in the shell were position restrained using a force constant of 300 kJ mol^-1^ nm^-1^. The system was subjected to equilibration for 200 ns.
3. Free equilibration: After equilibration in presence of “water-lined pores”, the atom types of modified lipids were reverted and the equilibration was continued for another 100 ns. Finally, the system was further equilibrated for another 125 ns without any position restraints.

The APL for vesicles is determined using a small patch of bilayer (14 nm × 14 nm) from the vesicle, assuming it to be flat. As the vesicle is large enough, we ignored the effects due to curvature. This bilayer patch was split into two individual monolayers and Voronoi tessellation was applied on each monolayer to obtain the APL for the monolayer [36]. The coordinates of PO4 (ROH) were used to define the position of each phospholipid (cholesterol) moiety.

For vesicle-protein system, a similar spontaneous aggregation procedure was followed as described above, for the vesicle. The CP lipids shell consisted of 525 POPS, 1659 POPE and 17976 CHOL molecules. The IV lipids shell consisted of 253 PPCS, 1230 POPC and 1010 CHOL molecules. The inner and outer diameters of IV lipids shell were 7 nm and 12 nm. The inner and outer diameters of CP lipids shell were 11.5 nm and 26.5 nm. In addition, twenty copies of Syb2 protein were randomly placed such that for each Syb2, the C-terminus (Thr116) lied in the IV shell, the Trp90 lied in the CP lipids shell and the SNARE motif lied at the exterior of the vesicle. The system was placed in a cubic box of ~48 nm, solvated with water and NA+ ions and energy minimized as above. The total production run was carried out for 197 ns.
